# Prenatal exposure to pesticides and risk for holoprosencephaly: a case-control study

**DOI:** 10.1186/s12940-020-00611-z

**Published:** 2020-06-08

**Authors:** Yonit A. Addissie, Paul Kruszka, Angela Troia, Zoë C. Wong, Joshua L. Everson, Beth A. Kozel, Robert J. Lipinski, Kristen M. C. Malecki, Maximilian Muenke

**Affiliations:** 1 Medical Genetics Branch, National Human Genome Research Institute, The National Institutes of Health, Bethesda, MD USA; 2 National Heart, Lung, and Blood Institute, The National Institutes of Health, Bethesda, MD USA; 3 Department of Comparative Biosciences, School of Veterinary Medicine, University of Wisconsin-Madison, Madison, WI USA; 4 Molecular and Environmental Toxicology Center, University of Wisconsin-Madison, Madison, WI USA; 5 Department of Population Health Sciences, School of Medicine and Public Health, University of Wisconsin-Madison, Madison, WI USA

**Keywords:** Pesticides, Holoprosencephaly, Forebrain defect, Environmental exposure, Prenatal exposure

## Abstract

**Background:**

Pesticide exposure during susceptible windows and at certain doses are linked to numerous birth defects. Early experimental evidence suggests an association between active ingredients in pesticides and holoprosencephaly (HPE), the most common malformation of the forebrain in humans (1 in 250 embryos). No human studies to date have examined the association. This study investigated pesticides during multiple windows of exposure and fetal risk for HPE. It is hypothesized that pre-conception and early pregnancy, the time of brain development in utero, are the most critical windows of exposure.

**Methods:**

A questionnaire was developed for this retrospective case-control study to estimate household, occupational, and environmental pesticide exposures. Four windows of exposure were considered: preconception, early, mid and late pregnancy. Cases were identified through the National Human Genome Research Institute’s ongoing clinical studies of HPE. Similarly, controls were identified as children with Williams-Beuren syndrome, a genetic syndrome also characterized by congenital malformations, but etiologically unrelated to HPE. We assessed for differences in odds of exposures to pesticides between cases and controls.

**Results:**

Findings from 91 cases and 56 controls showed an increased risk for HPE with reports of maternal exposure during pregnancy to select pesticides including personal insect repellants (adjusted odds ratio (aOR) 2.89, confidence interval (CI): 0.96–9.50) and insecticides and acaricides for pets (aOR 3.84, CI:1.04–16.32). Exposure to household pest control products during the preconception period or during pregnancy was associated with increased risk for HPE (aOR 2.60, OR: 0.84–8.68). No associations were found for occupational exposures to pesticides during pregnancy (aOR: 1.15, CI: 0.11–11.42), although exposure rates were low. Higher likelihood for HPE was also observed with residency next to an agricultural field (aOR 3.24, CI: 0.94–12.31).

**Conclusions:**

Observational findings are consistent with experimental evidence and suggest that exposure to personal, household, and agricultural pesticides during pregnancy may increase risk for HPE. Further investigations of gene by environment interactions are warranted.

## Background

Holoprosencephaly (HPE) is the most common malformation of the forebrain in humans. As with other birth defects, the etiology of HPE is complex with genetic and environmental factors thought to interact and interfere with forebrain development [1]. Characterized by failed or incomplete division of the prosencephalon, HPE has a prevalence of 1 in 250 early embryos [2]. The critical period for HPE is during gastrulation early in embryogenesis between the 18th and 28th days of gestation, when pregnancies are often not yet recognized. While relatively common in utero, HPE often results in spontaneous abortions and has a birth rate of 1 in 10,000 [3–5], making epidemiologic studies identifying non-genetic risk factors more difficult.

The genetic causes of HPE include chromosomal abnormalities [6], single genes associated with syndromes where HPE is a component [7], and genes associated with non-syndromic (isolated) cases where HPE is the only finding [6, 8, 9]. However, the etiology of many cases of HPE remains unknown with only 25% of patients with isolated HPE having mutations in one of the four most common genes associated with the condition [8, 10]. Furthermore, the genes associated with HPE are themselves characterized by incomplete penetrance and variable expressivity where family members who are obligate carriers of the same mutations as patients don’t have HPE or only show very mild features [8, 11]. Therefore, environmental risk factors are believed to be important contributors as modifiers of genetic mutations and/or direct insults in HPE. Early experimental evidence suggests that ingredients in pesticides may alter the HPE-associated Sonic Hedgehog (Shh) signaling pathway, possibly leading to an increased risk for holoprosencephaly [12].

Epidemiologic and animal studies suggest that the interactions between genetic and environmental factors underlie the etiologic heterogeneity and complexity of human birth defects [13], particularly HPE [1]. Human brain formation occurs early in gestation and is particularly vulnerable to perturbation by teratogens [14] including pesticides [15]. Prenatal exposure to pesticides has been related to increased risk for neural tube defects and anencephaly [15–17]. However, much less is known about related defects such as HPE that are often undetected in surveillance programs. No human studies to date have examined the relationships between pesticide exposures in pregnancy and HPE.

The strongest evidence for an environmental influence in HPE is found for maternal diabetes mellitus, which was associated with a 200-fold increase in the risk for HPE [18]. Evidence for other potential risk factors can be either inconsistent or, for factors like pesticides, simply lacking [19, 20]. Given the neurotoxic nature of many pesticides [21, 22], increased susceptibility of the developing brain to toxic agents [14], associations of pesticides with brain malformations [15–17], and experimental evidence linking ingredients in pesticides with inhibition of the most important pathway in holoprosencephaly [12], investigation of pesticide exposure in HPE etiology is warranted.

The low birth prevalence of HPE has hindered examination of potential non-genetic risk factors like pesticides in observational studies. Our group’s ongoing genetic studies on HPE at the National Human Genome Research Institute have allowed us to have the largest sample collection of patients with HPE in the world [11, 23]. Utilizing this unique resource, we conducted a case-control study aimed at determining if there is a relationship between prenatal exposures to pesticides and HPE. Previous case-control studies on HPE often relied on maternal recall of exposures in cases and healthy controls, which can be biased by an adverse outcome in study groups but a healthy outcome in control groups. In this study, our control group consisted of patients with Williams-Beuren syndrome (WBS), a congenital disorder with a known genetic etiology of a recurrent deletion in chromosome 7q11.23 [24]. Phenotype of Williams-Beuren syndrome includes characteristic facial features, congenital heart disease, intellectual disability and developmental delay [25]. The goal of including a non-typically developing control group was to account for retrospective bias and specifically the potential for reporting bias due to having a child with a congenital malformation.

## Methods

### Selection of cases and controls

Sample size was restricted by recruitment of cases and controls with rare genetic birth defects available via ongoing studies and registries. Only a few retrospective case-control studies have been done so far to investigate associations between HPE and maternal exposures gathered outside of vital statistics or during other administrative processes [2, 26–29], with this study being the first to investigate pesticide exposures. No a-prior data on effect sizes were available for this investigation. Cases were primarily ascertained from families of patients with HPE enrolled in our group’s clinical and genetic studies on HPE (clinicaltrials.gov: NCT00088426) as monitored by the National Human Genome Research Institute’s Institutional Review Board [11, 23]. For these studies, specimens and clinical data were gathered for each confirmed HPE patient. While the HPE study has been ongoing, the most recent pregnancies of patients with HPE (within 5 years) were emphasized for this study. A total of 92 cases were in the final study, 61 cases were ascertained through these studies while the remaining (*n* = 31) were ascertained through advertisements in websites and recruitment efforts with Families for HoPE, a nonprofit organization dedicated to address the needs of patients and families with HPE. Participants included those who tested negatively for four of the most common genes tested for HPE (*SHH, ZIC2, SIX3,* and *TGIF1*) and those with positive mutation status. Exclusion criteria for this group included cases where the patient’s etiology was known to be syndromic or chromosomal. The control group consisted of patients with Williams-Beuren syndrome (WBS). Participants (*n* = 56) were ascertained via advertisements through online platforms of the Williams Syndrome Association and study fliers. Nine of the participants were also enrolled in a National Heart, Lung, and Blood Institute study on WBS (clinicaltrials.gov: NCT02706639). Similar to cases, recruitment of the most recent pregnancies of patients (within the last 5 years) with WBS was prioritized. The recruitment period for new participants ran from March of 2016 to February of 2019. Participants were formally consented to take part in the study and signed a study consent form.

### Data collection

We designed a questionnaire focusing predominantly on maternal exposures including to pesticides during the prenatal period. The questionnaire was designed to gather information about risk factors for HPE more broadly, with pesticide exposures nested within it. The questionnaire was modified from the Early Life Exposures Assessment Tool (ELEAT) [30]. The ELEAT is a standardized instrument developed by Schmidt and Walker, and colleagues at the University of California-Davis as a systematic method for early life and pregnancy exposures for child development research, in particular autism. Core modules included demographics, pregnancy history, diet, lifestyle, and other environmental and occupational exposures. Questions regarding specific pesticide exposures were added to address unique study hypotheses. Formatting of the questionnaire was adapted for online and telephone-based interviews.

Pesticide exposures were estimated from a range of questions regarding a variety of potential sources including through use of pesticide-containing products by the mother or by anyone else in the household. Product use included personal insect repellants (spray, lotion, or towelettes); medications for lice or scabies; pest control products for pets to control for fleas, ticks and mites (including flea collars, flea and tick powders, shampoos, or other flea, tick and mite control products); products to control for pests or insects in or outside the home; weed killers used on the yard/lawn, flowers, vegetables, or fruit trees outside the home; and weed killers used on the flowers, vegetables, or fruit trees inside the home. We assessed occupational exposures to pesticides and residential proximity to an agricultural field (within 100 m). The questionnaire also elicited information about certain demographic characteristics and other exposures that could be protective or associated with risk for HPE including maternal intake of vitamins containing folic acid during pregnancy, and substance use including alcohol and cigarettes.

The questionnaire was also designed to examine different windows of exposure including 3 months prior to pregnancy, early (first trimester), mid (second trimester) and late (third trimester) pregnancy. Because women are often not aware of their pregnancies until a few weeks into the pregnancy, recall of the timing of exposures during early pregnancy may not be precise. For that reason, we also asked about exposures occurring during the 3 months before pregnancy (preconception period) as potentially relevant for HPE etiology. Questionnaires were primarily administered online on our study website in the National Institutes of Health’s Clinical Trials Database (CTDB). For five participants, the questionnaire was administered over the phone by trained interviewers.

### Statistical analysis

Both univariable and multiple logistic regression were used to examine associations between different types of pesticide exposures during different windows and HPE. For each pesticide exposure, we first used Fisher’s exact test and simple logistic regression (for non-dichotomous predictor variables) to test the independent effect of each factor on the risk of HPE occurrence. After a review of the literature, potential confounding variables were chosen based on exposure to pesticides and co-variates commonly used in studies on HPE [19, 20] that were assessed in this questionnaire. These variables included: maternal age, alcohol use, smoking, folic acid use during the preconception period and/or the first month of pregnancy, and time from estimated date of delivery to survey. The variables were each assessed separately using univariable logistic regression and, if they had a significant test, included as covariates in multivariable analyses. The threshold of significance for potential covariates was a *p* value of less than 0.05, or an odds ratio with confidence intervals not overlapping 1. The Haldane-Anscombe correction (adding 0.5 to all zero cells in the contingency table) was used to calculate odds ratios and corresponding confidence intervals [31]. Statistical analysis was conducted with R version 3.5.1 [32].

Sub-variable calculations, such as those on specific timeframe of exposure, were calculated after excluding participants who answered the main exposure question, but answered “don’t know” or did not provide an answer to timeframe questions. Observational studies of birth defects are often challenged by low case ascertainment and rare exposures leading to wide confidence intervals [19, 20]. Owing to the low birth prevalence of HPE, such results can even be seen in large, population-wide case-control studies. Nonetheless, estimated associations were interpreted with caution as needing further investigation.

## Results

Table 1 describes the study population including HPE cases and WBS controls. There were no significant differences between cases and controls. The proportion of female sex for fetuses and babies was slightly higher (59.5%) in cases compared to controls (45.8%) (*p* = 0.13). Approximately 38% of cases compared to 32% of controls lived in rural areas. Maternal age was comparable in the two groups while paternal age was higher in the control group. The majority of cases and controls were born to mothers with age < 35. In accordance with previous findings, among the HPE cases, 23.6% of tested probands had positive status for a mutation in one of the four most common genes associated with HPE, while maternal pregestational diabetes was found in 8 cases (9.2%) and 0 controls [10, 18]. Average time (years) since pregnancy and questionnaire was 3.58 for cases compared to 2.33 for controls. While the majority of study participants were from the U.S., other countries represented by cases included The United Kingdom, Canada, Pakistan, The Netherlands and Portugal while countries represented by controls included The United Kingdom, Canada, Sweden, Mexico and Australia.

**Table 1:** Description of Cases and Controls

Characteristic	Cases (%)	Controls (%)	***P***- Value^***a***^
Sex			0.13
Male	34 (40.5)	26 (54.2)	
Female	50 (59.5)	22 (45.8)	
Maternal age			0.80
< 35 years	55 (72.4)	36 (75.0)	
≥ 35	21 (27.6)	12 (25.0)	
Paternal age			0.31
< 35 years	49 (66.2)	28 (57.1)	
≥ 35	25 (33.8)	21 (42.9)	
Residential location			0.60
Urban	50 (58.8)	36 (66.7)	
Rural	32 (37.6)	17 (31.5)	
Both	3 (3.5)	1 (1.9)	
Alcohol consumption^*b*^			0.06
No	29 (38.2)	12 (22.2)	
Yes	47 (61.8)	42 (77.8)	
Before 3 months			
No	32 (41.6)	13 (24.1)	
Yes	45 (58.4)	41 (75.9)	
1st Trimester			
No	68 (89.5)	45 (83.3)	
Yes	8 (10.5)	9 (16.7)	
2nd Trimester			
No	74 (97.4)	50 (92.6)	
Yes	2 (2.6)	4 (7.4)	
3rd Trimester			
No	72 (94.7)	46 (85.2)	
Yes	4 (5.3)	8 (14.8)	
Smoking			0.28
No	68 (86.1)	50 (92.6)	
Yes	11 (13.9)	4 (7.4)	
Before 3 months			
No	68 (86.1)	50 (92.6)	
Yes	11 (13.9)	4 (7.4)	
1st Trimester			
No	73 (92.4)	52 (96.3)	
Yes	6 (7.6)	2 (3.7)	
2nd Trimester			
No	78 (98.7)	52 (96.3)	
Yes	1 (1.3)	2 (3.7)	
3rd Trimester			
No	78 (98.7)	52 (96.3)	
Yes	1 (1.3)	2 (3.7)	
Folic acid supplement intake^*c*^			0.08
No	4 (10.3)	1 (1.9)	
Yes	35 (89.7)	52 (98.1)	
Before 3 months			
No	21 (53.8)	18 (34.0)	
Yes	18 (46.2)	35 (66.0)	
1st month			
No	13 (37.1)	5 (10.4)	
Yes	22 (62.9)	43 (89.6)	
2nd month			
No	6 (17.1)	3 (6.2)	
Yes	29 (82.9)	45 (93.8)	
3rd month			
No	7 (20.0)	4 (8.3)	
Yes	28 (80.0)	44 (91.7)	
2nd trimester			
No	8 (22.9)	5 (10.4)	
Yes	27 (77.1)	43 (89.6)	
3rd trimester			
No	8 (22.9)	7 (14.6)	
Yes	27 (77.1)	41 (85.4)	
Time since delivery to survey (median years)	3.58	2.33	0.04
4-Gene Screen Status			
Positive (%)	13 (23.6)	NC	NC
SHH	2 (15.4)	NC	
ZIC2	6 (46.2)	NC	
SIX3	5 (38.5)	NC	
TGIF	0 (0.0)	NC	
Negative (%)	42 (76.4)	NC	

Note: *NC* Not calculated

^*a*^*P*-value based on Fischer’s exact test or simple logistic regression

^*b*^One drink = 1 12 oz. beer, 1 glass 4 oz. wine, or 1 oz. or 1 shot liquor (alone or in a mixed drink)

^*c*^Vitamin or supplement containing at least 400 mg folic acid or prenatal vitamin; intake during the three months before pregnancy and/or during pregnancy

Odds of HPE compared to WBS were assessed for each of the potential confounding variables separately (Table 2). Maternal use of folic acid containing supplements during the preconception period and/or during the first month of pregnancy was associated with reduced odds of HPE (odds ratio (OR) 0.24, confidence interval (CI): 0.06–0.86, *p* = 0.03). Time from delivery to survey completion was also significant (OR 1.06, CI: 1.00–1.14, *p* = 0.04). These two variables were subsequently included as covariates in all multivariable models.

**Table 2:** Univariate and Multiple Logistic Regression Analysis of Potential Risk Factors for Holoprosencephaly

Variable	OR (95%CI)	aOR (95%CI)^***a***^
Maternal age		
< 35 years	Ref	Ref
≥ 35	1.11 (0.49, 2.60)	0.76 (0.22, 2.49)
Alcohol consumption^*b*^		
No	Ref	Ref
Yes	0.47 (0.19, 1.08)	0.35 (0.11, 1.05)
Before 3 months		
No	Ref	Ref
Yes	0.45 (0.19, 1.02)	0.36 (0.12, 1.06)
1st Trimester		
No	Ref	Ref
Yes	0.59 (0.18, 1.87)	1.05 (0.23, 4.53)
2nd Trimester		
No	Ref	Ref
Yes	0.34 (0.03, 2.48)	0.52 (0.02, 6.65)
3rd Trimester		
No	Ref	Ref
Yes	0.32 (0.07, 1.29)	0.35 (0.04, 1.83)
Smoking		
No	Ref	Ref
Yes	2.01 (0.55, 9.18)	1.07 (0.17, 6.64)
Before 3 months		
No	Ref	Ref
Yes	2.01 (0.55, 9.18)	1.07 (0.17, 6.64)
1st Trimester		
No	Ref	Ref
Yes	2.13 (0.36, 22.35)	1.17 (0.14, 10.63)
2nd Trimester		
No	Ref	NC
Yes	0.34 (0.006, 6.61)	NC
3rd Trimester		
No	Ref	NC
Yes	0.34 (0.006, 6.61)	NC
Folic acid supplement intake^*c*^		
No	Ref	Ref
Yes	0.17 (0.008, 1.20)	0.19 (0.01, 1.42)
Before 3 months		
No	Ref	Ref
Yes	0.44 (0.19, 1.02)	0.46 (0.18, 1.13)^d^
1st month		
No	Ref	Ref
Yes	0.20 (0.06, 0.59)	0.27 (0.08, 0.86)^d^
2nd month		
No	Ref	Ref
Yes	0.32 (0.06, 1.32)	0.60 (0.10, 3.28)
3rd month		
No	Ref	Ref
Yes	0.36 (0.09, 1.32)	0.98 (0.19, 5.89)
2nd trimester		
No	Ref	Ref
Yes	0.39 (0.11, 1.30)	0.85 (0.20, 3.94)
3rd trimester		
No	Ref	Ref
Yes	0.58 (0.18, 1.78)	0.92 (0.26, 3.53)
Time since delivery to survey (median years)	1.06 (1.00, 1.14)	NC

Note: *OR* Crude odds ratio, *aOR* Adjusted odds ratio, *CI* Confidence Interval, *NC* Not calculated. Adjusted odds ratios for some variables (except for time since delivery to survey) were not calculated because of low rates of exposure to the potential risk factors and/or low ascertainment that did not permit estimations of the risk parameters

^*a*^ Models adjusted for maternal consumption of vitamin or supplement containing folic acid(at least 400 mcg during the 3 months before pregnancy and/or first month of pregnancy), and time since birth to completion of survey (continuous in years)

^*b*^One drink = 1 12 oz. beer, 1 glass 4 oz. wine, or 1 oz. or 1 shot liquor (alone or in a mixed drink)

^*c*^Vitamin or supplement containing at least 400 mg folic acid or prenatal vitamin; intake during the three months before pregnancy and/or during pregnancy

^*d*^Odds ratios are from logistic regression models adjusted for time since birth to survey completion

Table 3 provides a summary of self-report pesticide use during different windows of exposure from preconception through the third trimester of pregnancy. Of note, self-report of pesticide use for some common pesticides including those with N, N-diethyl-meta-toluamide (DEET) were rare.

Self-report of maternal exposure to personal insect repellants (through personal use or by anyone else in the household) during the preconception period was associated with increased odds for HPE (aOR 2.76, CI: 0.88–9.16), as was exposure during pregnancy (aOR 2.89, CI: 0.96–9.50) (Table 3). Within the group that used personal insect repellents, exposure to insect repellents containing DEET during the preconception period was associated with increased risk for HPE in an unadjusted model (OR 8.58, CI:1.03–185.33) and was similarly elevated but not statistically significant for exposure during pregnancy (OR 2.75, CI: 0.52–16.04). We could not assess risk after adjusting for confounders due to low numbers. Exposure rates for treatment for lice and scabies were similarly low and not associated with HPE risk (OR 1.62, CI: 0.06–42.44). Maternal and paternal occupational exposures to pesticides were not associated with HPE risk, although low exposure rates were found for these variables. Exposures to weed killers during the preconception period was not associated with HPE risk. While exposure rates were low, exposures to weed killers inside the home during pregnancy showed a trend towards association with HPE (aOR 5.15, CI: 0.65–107.01).

**Table 3:** Pesticide Exposures and Risk for Holoprosencephaly

Pesticide Exposure	Cases (%)	Controls (%)	OR (95%CI)	aOR (95%CI)^***a***^
Personal insect repellents^*b*^				
3 months before pregnancy				
No	22 (50.8)	30 (68.2)	Ref	Ref
Yes	32 (49.2)	14 (31.8)	2.08 (0.95, 4.71)	2.76 (0.88, 9.16)
Contained DEET				
No	1 (4.8)	4 (33.3)	Ref	NC
Yes	15 (71.4)	7 (58.3)	8.58 (1.03, 185.33)	NC
Used repellent with and without DEET	5 (23.8)	1 (8.3)	20.0 (1.32, 962.18)	NC
During pregnancy				
No	32 (50.8)	30 (65.2)	Ref	Ref
Yes	31 (49.2)	16 (34.8)	1.82 (0.84, 4.03)	2.89 (0.96, 9.50)
Entire pregnancy				
No	54 (90.0)	44 (95.7)	Ref	NC
Yes	6 (10.0)	2 (4.3)	2.44 (0.53, 17.25)	NC
First trimester				
No	50 (83.3)	42 (91.3)	Ref	Ref
Yes	10 (16.7)	4 (8.7)	2.10 (0.65, 8.10)	1.46 (0.25, 7.76)
Second trimester				
No	47 (78.3)	41 (89.1)	Ref	Ref
Yes	13 (21.7)	5 (10.9)	2.67 (0.78, 7.56)	4.18 (1.06, 18.95)
Third trimester				
No	49 (81.7)	39 (84.8)	Ref	Ref
Yes	11 (18.3)	7 (15.2)	1.25 (0.45, 3.68)	2.54 (0.70, 9.72)
Contained DEET				
No	4 (20.0)	5 (41.7)	Ref	NC
Yes	11 (55.0)	5 (41.7)	2.75 (0.52, 16.04)	NC
Used repellent with and without DEET	5 (25.0)	2 (16.7)	3.13 (0.42, 31.44)	NC
Weed killers outside home^*b*^				
3 months before pregnancy				
No	51 (60.7)	39 (73.6)	Ref	Ref
Yes	33 (29.3)	14 (26.4)	1.80 (0.86, 3.90)	1.81 (0.65, 5.10)
During pregnancy				
No	55 (64.7)	36 (69.2)	Ref	Ref
Yes	30 (35.3)	16 (30.8)	1.23 (0.59, 2.60)	1.66 (0.60, 4.67)
Weed killers inside home^*b*^				
3 months before pregnancy				
No	80 (95.2)	51 (96.2)	Ref	Ref
Yes	4 (4.8)	2 (3.8)	1.28 (0.24, 9.44)	0.76 (0.03, 8.86)
During pregnancy				
No	77 (90.6)	51 (98.1)	Ref	Ref
Yes	8 (9.4)	1 (1.9)	5.29 (0.93, 99.80)	5.15 (0.65, 107.01)
Treatments for lice and scabies^*b,c*^				
No	83 (96.5)	50 (98.0)	Ref	Ref
Yes	3 (3.5)	1 (2.0)	1.81 (0.22, 37.08)	1.62 (0.06, 42.44)
Pets inside home^*c*^				
No	24 (28.2)	20 (37.0)	Ref	Ref
Yes	61 (71.8)	34 (63.0)	1.50 (0.72, 3.09)	1.71 (0.62, 4.97)
Type				
Dog				
No	38 (44.7)	27 (50.0)	Ref	Ref
Yes	47 (55.3)	27 (50.0)	1.24 (0.62, 2.46)	1.15 (0.43, 3.06)
Cat				
No	58 (68.2)	38 (70.4)	Ref	Ref
Yes	27 (31.8)	16 (29.6)	1.11 (0.53, 2.35)	1.33 (0.45, 3.94)
Small mammal^*c*^				
No	80 (94.1)	51 (94.4)	Ref	Ref
Yes	5 (5.9)	3 (5.6)	1.06 (0.25, 5.36)	0.31 (0.01, 2.89)
Bird				
No	84 (98.8)	54 (1.00)	NC	NC
Yes	1 (1.2)	0 (0.0)	NC	NC
Fish or reptile				
No	79 (92.9)	50 (92.6)	Ref	Ref
Yes	6 (7.1)	4 (7.4)	0.95 (0.26, 3.87)	0.88 (0.11, 4.96)
Other				
No	84 (98.8)	53 (98.1)	Ref	Ref
Yes	1 (1.2)	1 (1.9)	0.63 (0.02, 16.18)	1.90 (0.07, 50.18)
Pesticides for pets^*b,c,e*^				
No	49 (64.5)	42 (79.2)	Ref	Ref
Yes	27 (35.5)	11 (20.8)	2.10 (0.95, 4.89)	3.13 (0.69, 6.75)
3 months before pregnancy				
No	55 (75.3)	43 (82.7)	Ref	Ref
Yes	18 (24.7)	9 (17.3)	1.56 (0.65, 3.97)	1.44 (0.38, 5.39)
During pregnancy				
No	52 (70.3)	45 (86.5)	Ref	Ref
Yes	22 (29.7)	7 (13.5)	2.72 (1.11, 7.42)	3.84 (1.04, 16.32)
First trimester				
No	58 (86.6)	46 (92.0)	Ref	Ref
Yes	9 (13.4)	4 (8.0)	1.78 (0.54, 6.92)	0.21 (0.009, 1.86)
Second trimester				
No	58 (86.6)	47 (94.0)	Ref	Ref
Yes	9 (13.4)	3 (6.0)	1.56 (0.83, 3.38)	1.02 (0.38, 2.61)
Third trimester				
No	59 (88.1)	47 (94.0)	Ref	Ref
Yes	8 (11.9)	3 (6.0)	1.29 (0.83, 2.16)	0.89 (0.42, 1.73)
Personally apply products for pets				
No	59 (80.8)	47 (90.4)	Ref	Ref
Yes	14 (19.2)	5 (9.6)	2.23 (0.79, 7.31)	1.22 (0.23, 6.05)
Regular encounter with horse^*c,f*^				
No	72 (96.0)	45 (97.8)	Ref	Ref
Yes	3 (4.0)	1 (2.2)	1.88 (0.23, 28.54)	1.80 (0.07, 47.50)
Fly repellents for horses				
No	8 (88.9)	4 (1.00)	Ref	NC
Yes	1 (11.1)	0 (0.0)	1.58 (0.05, 47.51)	NC
Pest problems^*c*^				
No	51 (63.0)	31 (63.3)	Ref	Ref
Yes	30 (37.0)	18 (36.7)	1.01 (0.49, 2.13)	0.66 (0.21, 1.98)
During entire pregnancy				
No	66 (81.5)	42 (87.5)	Ref	Ref
Yes	15 (18.5)	6 (12.5)	1.59 (0.60, 4.75)	1.47 (0.33, 6.43)
First trimester				
No	76 (93.8)	45 (93.8)	Ref	Ref
Yes	5 (6.2)	3 (6.2)	0.99 (0.23, 4.99)	0.15 (0.005, 1.90)
Second trimester				
No	77 (95.1)	42 (87.5)	Ref	NC
Yes	4 (4.9)	6 (12.5)	0.36 (0.09, 1.34)	NC
Third trimester				
No	79 (97.5)	44 (91.7)	Ref	NC
Yes	2 (2.5)	4 (8.3)	0.28 (0.04, 1.49)	NC
Household pest control products^*b,c,g*^				
No	36 (52.2)	31 (63.3)	Ref	Ref
Yes	33 (47.8)	18 (36.7)	1.58 (0.75, 3.37)	2.60 (0.84, 8.68)
3 months before pregnancy				
No	47 (75.8)	37 (77.1)	Ref	Ref
Yes	15 (24.2)	11 (22.9)	1.07 (0.44, 2.66)	1.99 (0.52, 7.80)
During pregnancy				
No	46 (71.9)	33 (71.7)	Ref	Ref
Yes	18 (28.1)	13 (28.2)	0.99 (0.43, 2.34)	2.16 (0.62, 7.78)
First trimester				
No	53 (82.8)	38 (82.6)	Ref	Ref
Yes	11 (17.2)	8 (17.4)	0.99 (0.36, 2.77)	2.06 (0.49, 8.43)
Second trimester				
No	53 (82.8)	36 (78.3)	Ref	Ref
Yes	11 (17.2)	10 (21.7)	0.86 (0.53, 1.40)	1.27 (0.63, 2.51)
Third trimester				
No	52 (81.2)	38 (82.6)	Ref	Ref
Yes	12 (18.8)	8 (17.4)	1.03 (0.74, 1.45)	1.28 (0.76, 2.11)
Occupational exposures to pesticides and herbicides				
Maternal: 3 months before pregnancy				
No	53 (86.9)	33 (94.3)	Ref	Ref
Yes	8 (13.1)	2 (5.7)	2.47 (0.45, 25.27)	1.95 (0.07, 52.19)
Maternal: during pregnancy				
No	54 (90.0)	31 (91.2)	Ref	Ref
Yes	6 (10.0)	3 (8.8)	1.15 (0.23, 7.58)	1.15 (0.11, 11.42)
Paternal^*c*^				
No	43 (72.9)	30 (85.7)	Ref	Ref
Yes	16 (27.1)	5 (14.3)	2.21 (0.68, 8.59)	2.39 (0.34, 20.58)
Living next to an agricultural field^*c,h*^				
No	70 (82.4)	47 (87.0)	Ref	Ref
Yes	15 (17.6)	7 (13)	1.44 (0.50, 4.49)	3.24 (0.94, 12.31)

Note: *OR* Crude odds ratio, *aOR* Adjusted odds ratio, *CI* Confidence Interval, *NC* Not calculated, *DEET* N, N-diethyl meta toluamide. Adjusted odds ratios for some variables were not calculated because of low rates of exposure to the potential risk factors and/or low ascertainment that did not permit estimations of the risk parameters

^a^All odds ratios are from logistic regression models adjusted for maternal consumption of vitamin or supplement containing folic acid(at least 400 mcg during the 3 months before pregnancy and/or first month of pregnancy), and time since birth to completion of survey (continuous in years)

^b^Use by mother or anyone in household

^c^During the 3 months before pregnancy and/or during pregnancy

^d^Rabbit, gerbil, hamster, guinea pig, ferret, mouse

^e^To control for fleas, ticks or mites

^f^Own, lease, train or care for horse regularly (once a week or more) 3 month before pregnancy until birth

^g^Products to control for pests or insects such as bugs like ants, wasps or others in or outside the home

^h^Within 100 m, e.g. field next door or across the street, during the 3 months before pregnancy or 1st trimester

While many other sources of pesticide use were rare, use of pest control products for pets or in the home were among the most significantly associated pesticides with increased odds of HPE compared to controls. Both the HPE and control groups were comparable in their likelihood of having pets (OR 1.71, CI: 0.62–4.97) and the types of pets found inside the home during the preconception period and during pregnancy. On the other hand, exposures during pregnancy to insecticides and acaricides (through maternal use or by anyone else in the household) on pets to control for fleas, ticks and mites was positively associated with the risk for HPE (OR 3.84, CI: 1.04–16.32). This did not depend on the mother personally applying the products for pets (OR: 1.22, CI: 0.23–6.05). Similarly, despite comparable reports of pest problems inside or outside the home in both cohorts (OR .66, CI:0.21–1.98), exposures to pest control products were associated with increased risk for HPE (OR: 2.60, CI:0.84–8.68). This association was not limited to exposures during a certain time period, but during both the preconception period and pregnancy.

Residential proximity to farms may also be another important source of pesticide exposure. Results showed that living next to an agricultural field (within 100 m, e.g. field next door or across the street) during the preconception period or early pregnancy (1st trimester) was positively associated with HPE risk (aOR 3.24, OR: 0.94–12.31).

## Discussion

The findings from this first case-control study of HPE and pesticide exposures demonstrate a significant relationship between prenatal exposure to pesticides and the potential risk for holoprosencephaly, the most common malformation of the forebrain in humans. Reports of maternal exposures to personal insect repellents during the preconception period and during pregnancy was positively associated with HPE with an observed two-fold increase in the risk for HPE. While mounting experimental and epidemiological evidence suggests a role of pesticides in the complex etiologies of birth defects [15, 33], little information is known regarding environmental exposures and HPE. To our knowledge, this is the first case control study investigating an association between pesticides and HPE. Previous epidemiological studies on HPE have evaluated factors like occupation and maternal state or country of residence during pregnancy but did not directly assess links between environmental exposures to pesticides and HPE [26, 27]. In this study, maternal and paternal occupational exposures to pesticides were not associated with HPE, although exposure rates were low especially for maternal exposures. On the other hand, we did find an increased risk for HPE with reports of living next to of an agricultural field. Additionally, there were marginal associations between exposures to herbicides inside the home during pregnancy and HPE, although exposure rates were low with a wide confidence interval.

Some of the factors that make pesticides potential teratogens in HPE is their neurotoxicity [21, 22], specific tendency to be more detrimental to the developing brain [34, 35] and associations with other birth defects [15, 33]. Accordingly, prenatal exposure to pesticides has been linked with other severe congenital anomalies of the brain such as anencephaly [15–17], which can entail microcephaly but is pathophysiologically and clinically different than HPE. Maternal exposures to pesticides have also been associated with orofacial clefts [36–38], which commonly co-occur with HPE [39] and have been linked to Sonic Hedgehog (Shh) signaling in animal models [40]. Adding to these links between pesticides and severe fetal anomalies, the findings from this study suggest that prenatal exposures to pesticides are associated with an increased risk for HPE.

A higher likelihood for HPE was observed with exposures to household pest control products and were also observed for exposures to insecticides and acaricides for pets. Interestingly, while exposures to pesticides for pets were associated with increased risk for HPE, when limiting the assessment to cases in which the mother directly applied these products, we found no differences in risk. These findings raise important questions about sources of pesticide exposures and suggest that risks are not limited to personal applications of the products by the mother but include exposures via product use by other people in the household that may result in increased pesticide residue in dust or other components of the home environment [41, 42]. Residential pesticide use has been shown to contribute to the persistence of higher than recommended quantities of pesticide residues in the indoor air and surfaces for as long as 2 weeks after a single application [43, 44].

The identity of the parent chemicals of the pesticides reported in the study or other component ingredients in the products were not assessed. Future epidemiologic and experimental work should investigate associations between specific pesticide products and chemicals because nontargeted analyses grouping different pesticides including innocuous chemicals could mask the role of those chemicals contributing to HPE. With regards to insecticides, there are five major classes of synthetic insecticides on the market since the 1940s: organochlorines, organophosphates, carbamates, pyrethroids, and neonicotinoids [45]. Each class has a different mechanism of action and mode of developmental neurotoxicity. More recently, pyrethroid-based insecticides have become more common than the previously popular organophosphate-based insecticides [46], especially with products used for pest eradication within the household [47, 48]. Given these trends, it would be especially imperative to examine possible associations between exposures to components of pyrethroid insecticide formulations and the risk for HPE. In this study, exposures to insect repellents with DEET during the preconception period was positively associated with increased risk for HPE in an unadjusted model. Although DEET is generally considered safe to use as directed during pregnancy [49], most of the evidence comes from animal models and more epidemiologic data is needed to investigate a possible association between DEET exposure and risk for HPE.

Although investigations into pesticides and HPE are in their early stages, there is some evidence showing that a co-ingredient in pyrethroid insecticide formulations could be associated with HPE. Specifically, in vitro assays showed that piperonyl butoxide (PBO), a pesticide synergist found in over a thousand pyrethroid insecticide formulations [50], inhibits the HPE-associated Sonic Hedgehog signaling pathway [12]. Another recent report illustrated that acute in utero PBO exposure targeting Shh signaling in forebrain development can cause HPE-associated abnormalities in the mouse [34]. PBO is one of the top 10 products detected in indoor dust [51] and may constitute as much as 4% of products directly applied to human skin [52]. In humans, one study found dose-responsive association between elevated maternal exposure to PBO during pregnancy and delayed neurocognitive development in the child [53]. Interestingly, this correlation was not observed for the active pesticide ingredient pyrethrin. But exposure assessment for the study focused on the third trimester or levels found in maternal and umbilical cord plasma after delivery. The associations observed in this study between insecticide exposures and HPE risk as well as the in vitro and in vivo assays demonstrating PBO as a Shh pathway inhibitor suggest that follow up studies are warranted to investigate the possible role of prenatal exposure to PBO on the risk of human HPE [12].

Dietary intake of pesticides, which could be a significant source of exposure [54, 55], was not explored in this study and should be investigated in future studies. In addition to epidemiological and animal studies investigating exposures to specific pesticides and co-ingredients, studies assessing pesticide biomarkers in serum during pregnancy would be ideal for not only identifying parent chemicals, but also ascertaining the magnitude of exposures to determine dose-dependent associations with risk for HPE. Future studies on HPE should also explore possible associations with exposures to pesticide mixtures, which have been related to increased risk for abnormal development including neural tube defects (as opposed to isolated exposures) [16, 56].

A major strength of this study is that it is, to our knowledge, the first well-characterized case-control study of HPE that uses a control group of a severe, but not pathophysiologically or etiologically related birth defect [6, 24, 39] as a control population. This study design is important to rule out potential recall bias inherent in retrospective studies for traumatic health outcomes including birth defects. The specific benefit of having a control group with a congenital anomaly can be seen considering the biased odds ratios that can result in self-report studies on exposures utilizing healthy control groups [57]. For example, Rull and colleagues [17] explored the association between residential proximity to agricultural crops during pregnancy and neural tube defects by comparing self-reports and land use maps of actual proximity. They found that case women were more likely to accurately recall living near agricultural crops than control women with healthy children. By having a control group with Williams-Beuren syndrome, we aimed to mitigate similar recall bias that depends on pregnancy outcome. Another strength of the study is the investigation of pesticide exposure through different routes including by use of different types of consumer products in the household, occupational exposures and exposures in the environment — largely yielding consistent associations with holoprosencephaly.

However, this study is not without important limitations. The results of this study should be interpreted carefully because multiple statistical tests were employed. Given the small sample size it is difficult to conclude causal associations, however, results of this analysis combined with emerging experimental data [12], increase potential weight of evidence that pesticide use and both active and inactive ingredients should be considered potential risk factors for HPE. It is also important to again note that some of the aforementioned associations had wide confidence intervals due to the small sample size and/or exposure rates. While the larger odds ratios suggest potentially important relationships, the wide confidence intervals necessitate further investigation of the findings in larger studies. At the same time, most previous studies of HPE report similarly large confidence intervals due to the challenges of case ascertainment and rarity of exposures under consideration. A total of 92 cases were identified in this study. The most recent study of environmental exposures to polycyclic aromatic hydrocarbons using data from the U.S. National Birth Defects Prevention Study includes 100 cases of HPE recruited over 13 years (1997–2011) [58]. The current study aims to provide a more contemporary and detailed exposure assessment which also extends previous studies in identifying a control group that has similar, but biologically unrelated neurodevelopmental defect. Thus, reporting bias between cases and controls should be minimized. This method, however, does limit power as both cases and controls were identified through unique registries and support groups for families of rare genetic birth defects.

Results should also be carefully considered because exposure assessment relied on retrospective reporting, which can be subject to both recall and reporting bias, particularly for severe birth defects such as HPE. The use of WBS controls was an attempt to control for the latter, and recall bias was hoped to be similarly skewed across both cases and controls which would tend to bias results towards the null. The results showed that women were more likely to report exposures across all windows of pregnancy but use of questionnaire data to assess specific windows was challenging using a retrospective design. One difference between the characteristics of cases and controls is the time since the delivery of the proband to survey completion, which was longer in the HPE cohort. The potential impact of this difference is that participants’ tendency to accurately recall their experiences during the time of their pregnancy may be differentially impaired in the two cohorts. To minimize the potentially confounding role of this difference, time since delivery to survey was included as a covariate in all multiple regression analyses. Despite these limitations, birth defects such as HPE remain an important public health and clinical challenge, leading to excessive fetal mortality and morbidity. Given the rarity and nature of birth defects as outcomes, a prospective study of preconception and early pregnancy outcomes is difficult and very costly. Therefore, this is a first look at potential associations that suggests areas for future research including prospective designs and the use of biomarkers for exposure. The significant strengths combined with these limitations suggest that further investigations using larger, prospective study designs should be considered.

## Conclusions

Despite the considerable public health burden of HPE, investigation of environmental factors has been extremely limited. Unique in its use of a control group affected by a congenital malformation, this study found that the previously unreported exposure of pesticides may be a risk factor for having a pregnancy affected by HPE. Further investigations should include biomarker studies, animal experiments and studies of gene by environment interactions.

## Data Availability

The data that support the findings of this study are available on request from the corresponding authors [PK and MM]. The data are not publicly available due to them containing information that could compromise research participant privacy/consent.

## Abbreviations

aOR: Adjusted odds ratio
DEET: N, N-diethyl-meta-toluamide
CI: Confidence interval
CTDB: Clinical trials database
HPE: Holoprosencephaly
OR: Odds ratio
Shh: Sonic Hedgehog
WBS: Williams-Beuren syndrome

## References

[1] Krauss RS, Hong M (2016). Gene-environment interactions and the etiology of birth defects. Curr Top Dev Biol.

[2] Matsunaga E, Shiota K (1977). Holoprosencephaly in human embryos: epidemiologic studies of 150 cases. Teratology..

[3] Leoncini E, Baranello G, Orioli IM, Anneren G, Bakker M, Bianchi F (2008). Frequency of holoprosencephaly in the international clearinghouse birth defects surveillance systems: searching for population variations. Birth Defects Res Part A Clin Mol Teratol.

[4] Orioli IM, Castilla EE, Ming JE, Nazer J, Burle de Aguiar MJ, Llerena JC (2001). Identification of novel mutations in SHH and ZIC2 in a south American (ECLAMC) population with holoprosencephaly. Hum Genet.

[5] Yi L, Liu Z, Deng C, Li X, Wang K, Deng K (2019). Epidemiological characteristics of holoprosencephaly in China, 2007-2014: a retrospective study based on the national birth defects surveillance system. PLoS One.

[6] Kruszka P, Martinez AF, Muenke M (2018). Molecular testing in holoprosencephaly. Am J Med Genet C Semin Med Genet..

[7] Kruszka P, Muenke M (2018). Syndromes associated with holoprosencephaly. Am J Med Genet C Semin Med Genet..

[8] Roessler E, Hu P, Muenke M (2018). Holoprosencephaly in the genomics era. Am J Med Genet C Semin Med Genet.

[9] Kruszka P, Berger SI, Weiss K, Everson JL, Martinez AF, Hong S (2019). A CCR4-NOT transcription complex, subunit 1, CNOT1, variant associated with Holoprosencephaly. Am J Hum Genet.

[10] Roessler E, Muenke M (2010). The molecular genetics of holoprosencephaly. Am J Med Genet C Semin Med Genet.

[11] Solomon BD, Mercier S, Velez JI, Pineda-Alvarez DE, Wyllie A, Zhou N (2010). Analysis of genotype-phenotype correlations in human holoprosencephaly. Am J Med Genet C Semin Med Genet.

[12] Wang J, Lu J, Mook RA, Zhang M, Zhao S, Barak LS (2012). The insecticide synergist piperonyl butoxide inhibits hedgehog signaling: assessing chemical risks. Toxicol Sci.

[13] Zhu H, Kartiko S, Finnell RH (2009). Importance of gene-environment interactions in the etiology of selected birth defects. Clin Genet.

[14] Dobbing J (1968). Applied neurochemistry: Blackwell scientific.

[15] Kalliora C, Mamoulakis C, Vasilopoulos E, Stamatiades GA, Kalafati L, Barouni R (2018). Association of pesticide exposure with human congenital abnormalities. Toxicol Appl Pharmacol.

[16] Rull RP, Ritz B, Shaw GM (2006). Neural tube defects and maternal residential proximity to agricultural pesticide applications. Am J Epidemiol.

[17] Rull RP, Ritz B, Shaw GM (2006). Validation of self-reported proximity to agricultural crops in a case-control study of neural tube defects. J Expo Sci Environ Epidemiol.

[18] Barr M, Hanson JW, Currey K, Sharp S, Toriello H, Schmickel RD (1983). Holoprosencephaly in infants of diabetic mothers. J Pediatr.

[19] Johnson CY, Rasmussen SA (2010). Non-genetic risk factors for holoprosencephaly. Am J Med Genet C Semin Med Genet.

[20] Summers AD, Reefhuis J, Taliano J, Rasmussen SA (2018). Nongenetic risk factors for holoprosencephaly: an updated review of the epidemiologic literature. Am J Med Genet C Semin Med Genet..

[21] Ross SM, McManus IC, Harrison V, Mason O (2013). Neurobehavioral problems following low-level exposure to organophosphate pesticides: a systematic and meta-analytic review. Crit Rev Toxicol.

[22] Mostafalou S, Abdollahi M (2013). Pesticides and human chronic diseases: evidences, mechanisms, and perspectives. Toxicol Appl Pharmacol.

[23] Roessler E, Hu P, Marino J, Hong S, Hart R, Berger S (2018). Common genetic causes of holoprosencephaly are limited to a small set of evolutionarily conserved driver genes of midline development coordinated by TGF-beta, hedgehog, and FGF signaling. Hum Mutat.

[24] Morris CA, Adam MP, Ardinger HH, Pagon RA, Wallace SE, LJH B, Stephens K (2017). Williams Syndrome. GeneReviews((R)).

[25] Kruszka P, Porras AR, de Souza DH, Moresco A, Huckstadt V, Gill AD (2018). Williams-Beuren syndrome in diverse populations. Am J Med Genet A.

[26] Miller EA, Rasmussen SA, Siega-Riz AM, Frias JL, Honein MA (2010). Risk factors for non-syndromic holoprosencephaly in the National Birth Defects Prevention Study. Am J Med Genet C Semin Med Genet.

[27] Croen LA, Shaw GM, Lammer EJ (2000). Risk factors for cytogenetically normal holoprosencephaly in California: a population-based case-control study. Am J Med Genet.

[28] Orioli IM, Amar E, Bakker MK, Bermejo-Sanchez E, Bianchi F, Canfield MA (2011). Cyclopia: an epidemiologic study in a large dataset from the International Clearinghouse of Birth Defects Surveillance and Research. Am J Med Genet C Semin Med Genet.

[29] Vaz SS, Chodirker B, Prasad C, Seabrook JA, Chudley AE, Prasad AN (2012). Risk factors for nonsyndromic holoprosencephaly: a Manitoba case-control study. Am J Med Genet A.

[30] Schmidt JR, Walker CK, Bennett D, Tancredi D (2019). Early Life Exposures Assessment Tool (ELEAT).

[31] Lawson R (2004). Small sample confidence intervals for the odds ratio. Commun Stat Simul Comput.

[32] Team RC (2013). R: a language and environment for statistical computing.

[33] Weselak M, Arbuckle TE, Foster W (2007). Pesticide exposures and developmental outcomes: the epidemiological evidence. J Toxicol Environ Health B Crit Rev.

[34] Shafer TJ, Meyer DA, Crofton KM (2005). Developmental neurotoxicity of pyrethroid insecticides: critical review and future research needs. Environ Health Perspect.

[35] Infants CoPitDo, Council CNR (1993). Pesticides in the diets of infants and children: National Academy Press.

[36] Romitti PA, Herring AM, Dennis LK, Wong-Gibbons DL (2007). Meta-analysis: pesticides and orofacial clefts. Cleft Palate Craniofac J.

[37] Yang W, Carmichael SL, Roberts EM, Kegley SE, Padula AM, English PB (2014). Residential agricultural pesticide exposures and risk of neural tube defects and orofacial clefts among offspring in the San Joaquin Valley of California. Am J Epidemiol.

[38] Spinder N, Bergman JEH, Boezen HM, Vermeulen RCH, Kromhout H, de Walle HEK (2017). Maternal occupational exposure and oral clefts in offspring. Environ Health.

[39] Solomon BD, Gropman A, Muenke M, Adam MP, Ardinger HH, Pagon RA, Wallace SE, LJH B, Stephens K (2013). Holoprosencephaly Overview. GeneReviews((R)).

[40] Heyne GW, Melberg CG, Doroodchi P, Parins KF, Kietzman HW, Everson JL (2015). Definition of critical periods for hedgehog pathway antagonist-induced holoprosencephaly, cleft lip, and cleft palate. PLoS One.

[41] Stout DM, Bradham KD, Egeghy PP, Jones PA, Croghan CW, Ashley PA (2009). American healthy homes survey: a national study of residential pesticides measured from floor wipes. Environ Sci Technol.

[42] Whyatt RM, Camann DE, Kinney PL, Reyes A, Ramirez J, Dietrich J (2002). Residential pesticide use during pregnancy among a cohort of urban minority women. Environ Health Perspect.

[43] Fenske RA, Black KG, Elkner KP, Lee CL, Methner MM, Soto R (1990). Potential exposure and health risks of infants following indoor residential pesticide applications. Am J Public Health.

[44] Gurunathan S, Robson M, Freeman N, Buckley B, Roy A, Meyer R (1998). Accumulation of chlorpyrifos on residential surfaces and toys accessible to children. Environ Health Perspect.

[45] Abreu-Villaça Y, Levin ED. Developmental neurobehavioral neurotoxicity of insecticides. In: Slikker W, Paule MG, Wang C, editors. Handbook of developmental neurotoxicology. Second edition. ed. London: Academic Press, an imprint of Elsevier; 2018. p. 453–66.

[46] Grube AH, Donaldson D, Kiely T (2004). Pesticides industry sales and usage: 2000 and 2001 market estimates: biological and economic analysis division, US Environmental Protection Agency.

[47] Williams MK, Rundle A, Holmes D, Reyes M, Hoepner LA, Barr DB (2008). Changes in pest infestation levels, self-reported pesticide use, and permethrin exposure during pregnancy after the 2000-2001 U.S. Environmental Protection Agency restriction of organophosphates. Environ Health Perspect.

[48] Power LE, Sudakin DL (2007). Pyrethrin and pyrethroid exposures in the United States: a longitudinal analysis of incidents reported to poison centers. J Med Toxicol.

[49] Wylie BJ, Hauptman M, Woolf AD, Goldman RH (2016). Insect repellants during pregnancy in the era of the Zika virus. Obstet Gynecol.

[50] List B (2006). Eligibility decision for Piperonyl Butoxide (PBO).

[51] Rudel RA, Camann DE, Spengler JD, Korn LR, Brody JG (2003). Phthalates, alkylphenols, pesticides, polybrominated diphenyl ethers, and other endocrine-disrupting compounds in indoor air and dust. Environ Sci Technol..

[52] Meinking TL, Serrano L, Hard B, Entzel P, Lemard G, Rivera E (2002). Comparative in vitro pediculicidal efficacy of treatments in a resistant head lice population in the United States. Arch Dermatol.

[53] Horton MK, Rundle A, Camann DE, Boyd Barr D, Rauh VA, Whyatt RM (2011). Impact of prenatal exposure to piperonyl butoxide and permethrin on 36-month neurodevelopment. Pediatrics..

[54] Chen M, Tao L, McLean J, Lu C (2014). Quantitative analysis of neonicotinoid insecticide residues in foods: implication for dietary exposures. J Agric Food Chem.

[55] Tanaka T, Inomata A (2015). Effects of maternal exposure to Piperonyl Butoxide (PBO) on behavioral development in F1-generation mice. Birth Defects Res B Dev Reprod Toxicol.

[56] Greenlee AR, Ellis TM, Berg RL (2004). Low-dose agrochemicals and lawn-care pesticides induce developmental toxicity in murine preimplantation embryos. Environ Health Perspect.

[57] Avruskin GA, Meliker JR, Jacquez GM (2008). Using satellite derived land cover information for a multi-temporal study of self-reported recall of proximity to farmland. J Expo Sci Environ Epidemiol.

[58] Santiago-Colon A, Rocheleau CM, Chen IC, Sanderson W, Waters MA, Lawson CC (2020). Association between maternal occupational exposure to polycyclic aromatic hydrocarbons and rare birth defects of the face and central nervous system. Birth Defects Res.

